# Risk Factors of Ventilator-Associated Pneumonia in Critically III Patients

**DOI:** 10.3389/fphar.2019.00482

**Published:** 2019-05-09

**Authors:** Diling Wu, Chenfang Wu, Siye Zhang, Yanjun Zhong

**Affiliations:** ^1^ICU Center, The Second Xiangya Hospital, Central South University, Changsha, China; ^2^Department of Surgery, University of Pittsburgh School of Medicine, Pittsburgh, PA, United States

**Keywords:** ventilator-associated pneumonia, mechanical ventilation, nosocomial infection, risk factor, intensive care unit, prevention

## Abstract

Ventilator-associated pneumonia (VAP), a hospital acquired pneumonia that occurs more than 48 h after mechanical ventilation, is a common complication of mechanical ventilation with a high mortality rate. VAP can cause patients to have difficulty weaning off the ventilator and to stay in the hospital longer, which results in a huge financial burden to patients and a huge demand for medical resources. Several strategies, such as drugs including chlorhexidine, β-lactam antibiotics and probiotics, have been used to prevent VAP in clinic. The incidence and the mortality rate of VAP have been decreased with the development of preventative strategies in the past decades, but VAP remains one of the most common causes of nosocomial infections and death in the intensive care unit. Current challenges in the management of VAP involved the lack of a gold standard for diagnosis, the absence of effective preventative strategies, and the rise in antibiotic resistance. Therefore, in order to reduce the incidence of VAP and improve the outcome of patients with mechanical ventilation, it is necessary to clarify the risk factors of VAP for clinical prevention and control of VAP. This paper reviews the international risk factors of VAP occurrence reported in recent years, including patient characteristics, increased mechanical ventilation time and prolonged length of hospital stay, disorders of consciousness, burns, comorbidities, prior antibiotic therapy, invasive operations, gene polymorphisms, and mentions the corresponding preventive measures. Each factor is not only an independent risk factor of VAP, but also has an influence on each other. A better understanding of risk factors for VAP is helpful for predicting the occurrence of VAP, improving the prevention and control of VAP, and reducing the morbidity and mortality rates of patients with VAP.

## Introduction

Mechanical ventilation is an effective intervention method to save the life of critically ill patients and is widely used in intensive care units (ICUs). Ventilator-associated pneumonia (VAP) is a type of nosocomial infections and occurs after more than 48 h of mechanical ventilation. The 2016 clinical guidelines released by the Infectious Diseases Society of America (IDSA) and the American Thoracic Society (ATS) reported that the mortality rate of VAP in the United States reached up to 13% (Kalil et al., 2016). While in Europe, a multi-center prospective study reported that the 30-day mortality rate of VAP was 29.9%, the mortality rate of early VAP was 19.2%, and the mortality rate of the late VAP was 31.4% (Martin-Loeches et al., 2018). A meta-analysis which included 8282 cases from 20 provinces of China indicated that the cumulative incidence of VAP in mainland China was 23.8% from 2006 to 2014 (Ding et al., 2017). There are several strategies used to prevent and control VAP in clinic, such as prevention bundles and drugs including chlorhexidine, β-lactam antibiotics and probiotics. Although the prevalence of VAP has declined in recent years due to the implementation of therapeutic strategies, it remains one of the most common causes of nosocomial infections and death of critically ill patients during hospitalization in ICUs. VAP can cause patients to have difficulty to get off the ventilator and to stay in the hospital longer, which cause a huge financial burden to patients and a huge demand for medical resources. Therefore, it is very important to clarify the risk factors of VAP to know more and get better prevention and control of VAP. As shown in Table 1, there are various risk factors for VAP. Patients’ characteristics (e.g., advanced age, male), increased mechanical ventilation time and prolonged mechanical ventilation, disorders of consciousness, burns, comorbidities, prior antibiotic therapy, invasive operations, and gene polymorphisms are the internationally recognized factors at present.

**TABLE 1:** Specific preventive measures for the occurrence of various risk factors of ventilator-associated pneumonia (VAP).

**Risk Factors**	**Preventive Measures**
Patient characteristics: Advanced age, Male	Not available
Increased mechanical ventilation time and prolonged length of hospital stay	Consider NIPPV* in certain types of respiratory failure, pre-intubation, or as weaning modalities;
	Spontaneous breathing trial;
	Daily awakening trial
Disorders of consciousness	Head of bed elevation to 30–45 degrees; Reducing unnecessary sputum suction;
	Reducing the replacement of ventilatory circuit;
	Early prophylactic use of antibiotics
Burns	Spontaneous breathing trial;
	Daily awakening trial;
	Limited liquid infusion
Comorbidities	Nutritional support;
	Preventive use of immunoglobulin;
	Rational use of antibiotics
Prior antibiotic therapy	Reduce the use of unnecessary antibiotics;
	Rational use of antibiotics
Invasive operations	Hand hygiene of medical staff;
	Silver-coated endotracheal tubes; Subglottic secretion drainage;
	Short course of intravenous antibiotic;
	Selective decontamination of the digestive tract
Gene polymorphisms	Early genetic identification
Other factors: Smoking, Intra- abdominal hypertension, and Hyperoxemia	Quit smoking;
	Head of bed elevation to 30–45 degrees
	Selective decontamination of the digestive tract etc.

NIPPV*: Non-Invasive Positive Pressure Ventilation.

## Patient Characteristics

A retrospective study of 147 patients aged 19–98 years showed that the average age of patients with VAP was 69.9 ± 15.9 years (But et al., 2017). Some clinical trials have also confirmed that advanced age (≥60 years) was an independent risk factor for susceptibility to VAP (Chang et al., 2017; Ding et al., 2017). Liu et al. (2017) reported that the likelihood of VAP increased by more than 1.15-fold per 1-year increase in age. The causes may be the decline of physiological function of respiration, the gradual atrophy of respiratory muscles, the gradual reduction of lung tissue elasticity, visibly weakened protective cough reflex and the decreased immune function in the elderly. The elderly are also often plagued by severe chronic illnesses and long-term malnutrition. Therefore, they are more susceptible to infection than young people. Infection in the elderly is associated with more clinical complications and a worse prognosis. A study reported that elderly people were prone to chronic basic diseases such as chronic heart failure, potential respiratory diseases, chronic renal failure, diabetes, and more non-metastatic cancers (Blot et al., 2014). However, another study demonstrated that there was no statistically significant difference in age between VAP and non-VAP groups, and that age was not a risk factor for VAP, but an independent risk factor for VAP mortality (Zubair et al., 2018).

A prospective cohort study of 747 patients reported that male gender is also an independent risk factor for VAP (Bornstain et al., 2004). Forel et al. (2012) performed a prospective study of 339 patients with severe acute respiratory distress syndrome (ARDS) and found that 98 (28.9%) had at least one VAP episode. The proportion of male patients in the VAP group was significantly higher than that in the non-VAP group, suggesting that gender was an independent risk factor for VAP. Tejerina et al. (2006) surveyed 2,897 patients in 361 ICUs in 20 countries and found that the relative risk of VAP in men was 1.3-fold more than that in women. A survey of 854 post-traumatic VAP patients by Sharpe et al. (2014) showed that 676 (79%) of them were men. Multivariate logistic regression analysis confirmed that gender was independently associated with susceptibility to VAP. Cui et al. (2018) suggested that male gender was associated with an increased risk of post-traumatic infection. Male is an independent risk factor for VAP in patients with subarachnoid hemorrhage. Differences in risk of VAP between men and women may be related to differences in sex hormones, effects of gender-related gene polymorphisms on drug immune responses, differences in the distribution of infectious pathogens between men and women, and differences in complications between men and women (Forel et al., 2012).

## Increased Mechanical Ventilation Time and Prolonged Length of Hospital Stay

Mechanical ventilation is an effective intervention method to save the life of critically ill patients and is widely used in ICUs. However, prolonged mechanical ventilation can lead to increased risk of infection and a variety of complications. VAP is a common complication of mechanical ventilation. In a 2017 retrospective study, it was reported that ventilation time and ICU length of stay were significantly longer in patients with VAP than in those without VAP (Abdelrazik and Salah Abdelazim, 2017). Mechanical ventilation for more than 2 weeks was a risk factor for VAP in ICU patients (Blot et al., 2014; Ding et al., 2017; Liu et al., 2017). An Egyptian study found that the incidence of VAP increased from 5% of patients receiving 1 day of mechanical ventilation to 65% of patients receiving 30 days of mechanical ventilation (Abdelrazik and Salah Abdelazim, 2017). The artificial airway established by mechanical ventilation changes the mucosal defense function of the normal airway. It weakens the ability of swallowing and the scavenging capacity of cilia to mucus. Bacteria directly enter the lower respiratory tract or pass through the gap between the tracheal tube wall and the airway leading to infection. In addition, long-term ventilation increases the risk of infection, which is caused by humidifiers and ventilator loops that are the source of the pathogen due to exposure (Ding et al., 2017). The majority of ICUs are closed units and air circulation is not smooth. The exhaled gasses and secretions of ICU patients contain a large number of pathogens causing air contamination. The chance of obtaining hospital acquired infections is also increased with extension of hospital stay.

## Disorders of Consciousness

Ventilator-associated pneumonia is a common complication in coma patients and may lead to poor prognosis. A study of 100 patients with VAP in Zagazig University showed that the Glasgow coma score in VAP group were significantly lower than that in non-VAP patients. The disorders of consciousness were significantly correlated with the occurrence of early VAP (Jovanovic et al., 2016; Chang et al., 2017; Othman et al., 2017). A study in Serbia in 2015 showed that the incidence of VAP patients with severe head injury was up to 49.7%, which was much higher than the average incidence of VAP. Moreover, pre-admission coma was an independent risk factor for late VAP (Jovanovic et al., 2015). A single-center retrospective study involving 200 patients with subarachnoid hemorrhage requiring mechanical ventilation from 2011 to 2015 showed a significant association between persistent sedation and VAP (Cui et al., 2018). Physiological reflexes of patients with disorders of consciousness, such as swallowing, coughing and expectoration, are weakened which affect the discharge of respiratory secretions. Additionally, patients with disorders of consciousness are in a passive position; they cannot cooperate with medical staff well to do some clinical operations. Therefore, gastric contents may reflux and cause aspiration in the process of passive sputum suction.

## Burns

The most significant clinical complication after burn is pneumonia, with an incidence of 10–65% (Latenser et al., 2007). A 5-year study of 92 burn children admitted to the pediatric ICU (PICU) showed that 57% of children had at least one diagnosis and treatment of pneumonia during their clinical course, of which 41% met the diagnostic criteria for VAP (Rogers et al., 2014). A prospective study of 80 burn patients by Mgahed et al. (2013) showed that the incidence of pneumonia in burn patients was 15%, and the incidence of pneumonia in the group with inhalational injury was twice as high as that in the group without inhalational injury. Sen et al. (2016) retrospectively studied 314 adult burn patients requiring mechanical ventilation. The results showed that the occurrence of VAP was 18%, accounting for 74% of total pneumonia. Compared with non-VAP patients, the burn area and inhalation injury were significantly increased in VAP patients. Multivariate logistic regression analysis showed that total body surface area (TBSA) was an independent risk factor for VAP in burn patients (Sen et al., 2016). A retrospective study of 163 burn patients showed that burn patients with moderate and severe International Normalized Ratio (INR) elevations (1.4–1.6 and >1.6, respectively) received longer mechanical ventilation, longer ICU lengths stay and longer hospital lengths of stay than those with normal INR. This suggested that early coagulopathy was significantly related to the incidence of ventilator-associated events (VAE) (Younan et al., 2017). The reasons may be increased vascular permeability and leakage of cytokines and proteins into the lung parenchyma resulting from severe burn, leading to direct lung injury. Severe burn may lead to acute respiratory failure and impaired bacterial clearance in the lungs. Severe burn may also result in significant immune dysfunction, potentially reducing the ability of the immune system to effectively eliminate bacteria. In addition, the contamination of burn wounds can cause hematogenous infection. Collectively, these may all increase the incidence of VAP (Rogers et al., 2014; Sen et al., 2016).

## Comorbidities

Chronic diseases might be a risk factor for VAP, including coronary disease, diabetes, respiratory diseases, chronic renal failure, and Hashimoto’s thyroiditis (But et al., 2017; Chang et al., 2017; Jimenez-Trujillo et al., 2017). In general, chronic diseases occur mostly in elderly patients and are usually accompanied by more than one comorbidity. These diseases together lead to immune suppression, causing impairments of vital organs such as heart, liver, kidney, and lungs, making the patient more vulnerable to infection. Patients with chronic diseases had longer mechanical ventilation time and hospital stay (Fabbri et al., 2004). Liu et al. (2017) further showed that patients with chronic obstructive pulmonary disease (COPD) were 2.35 times more likely to develop VAP than patients without COPD. COPD could be an independent predictor of VAP. Michetti et al. (2017) and Arumugam et al. (2018) confirmed that VAP patients had a higher Injury Severity Score (ISS) and Abbreviated Injury Score (AIS) of head/neck injuries greater than two. In addition, Younan et al. (2016) have shown that spinal cord injury is an independent predictor of VAP development. The reason might be that coagulopathy caused by early trauma could increase the risk of nosocomial infection.

## Prior Antibiotic Therapy

The common pathogens of VAP are Gram-negative bacilli including *Pseudomonas aeruginosa*, *Escherichia coli*, *Klebsiella pneumoniae*, and *Acinetobacter*, and Gram-positive cocci such as *Staphylococcus aureus*. *Pseudomonas aeruginosa* is the most common pathogen of VAP (Evans et al., 2018; Rhodes et al., 2018). In recent years, due to previous antibiotic treatment, the number of multidrug-resistant pathogens is increasing. The analysis of VAP etiology in a tertiary hospital indicated that *Enterobacteriaceae* and *Staphylococcus aureus* were the main pathogens in early VAP, non-fermenting bacteria and *Enterobacteriaceae* were the main pathogens in late VAP, and 60.8% of bacterial pathogens were multidrug-resistant strains. The study included 100 patients with clinically diagnosed VAP, 35 of whom were diagnosed with VAP by quantitative culture of endotracheal aspirates. Previous antibiotic therapy and hospitalization for more than 5 days were independent risk factors for drug resistance in VAP pathogens, according to risk factor assessments (Mahapatra et al., 2018). In addition, an etiological study of 397 VAP patients also showed that long-term exposure to unnecessary antibiotics was one of the strongest predictors of antibiotic resistance. Multivariate logistic analysis identified prophylactic antibiotic exposure and multiple inadequate antibiotic therapies as independent risk factors for multidrug-resistant VAP (Lewis et al., 2018). Although the distribution of ICU pathogens varies with patient populations, diagnostic methods, length of hospital stay and antibiotic policy, the growth trend of multidrug-resistant strains is consistent. Of course, there have been numerous reports that prophylactic use of antibiotics can reduce the incidence and mortality of VAP, but it is undeniable that long-term prophylactic use of antibiotics can lead to drug resistance changes in pathogens, making treatment more difficult. This is a question about time; the best time frame to use prophylactic antibiotics needs to be verified by more clinical trials.

## Invasive Operations

In the ICU, nosocomial infection is associated with invasive mechanical ventilation which includes re-intubation, tracheostomy and other operations (Horan et al., 2008). Walaszek et al. (2016) found that invasive medical treatments including re-intubation, tracheostomy and fiberoptic bronchoscopy were significant risk factors for VAP in the ICU (Walaszek et al., 2016). Previous studies have also shown that re-intubation, endotracheal sputum aspiration, fiberoptic bronchoscopy, tracheostomy and indwelling nasogastric tube were significantly associated with VAP (Apostolopoulou et al., 2003; Yuan et al., 2007; Thatrimontrichai et al., 2017). Among these, tracheostomy and re-intubation were independent risk factors for late VAP (Joseph et al., 2009; Othman et al., 2017). Giard et al. (2008) found that exposure to venous catheters before VAP was an independent risk factor for VAP in a comparison between early and late VAP. Increased intubation time, endotracheal sputum aspiration and fiberoptic bronchoscopy can destroy the normal epiglottic barrier and increase the likelihood of aspiration. In addition, the introduction of a large amount of bronchoalveolar lavage fluid could reduce the bacterial clearance rate (Apostolopoulou et al., 2003). Indwelling gastric tubes can damage the function of the cardiac sphincter and increase the chance of gastric fluid reflux, resulting in aspiration. Bacteria colonized in the stomach can be colonized in the pharynx through the stomach’s anti-peristaltic action and/or along the gastric tube, and then get into the lower respiratory tract to cause infection. Pulmonary parenchymal injury caused by pneumothorax or hemothorax after thoracic intubation could play an important role in the development of VAP according to a prospective study (Apostolopoulou et al., 2003).

## Gene Polymorphisms

The results of various studies on the effect of single nucleotide polymorphisms (SNPs) in the promoter region of tumor necrosis factor α (TNFα) gene on susceptibility to infection are controversial. Some suggest that patients carrying these SNPs alleles are more likely to become infected than those carrying only wild-type alleles (Elahi et al., 2009; Read et al., 2009). Recently, a study showed that any A allele of SNP at 376, 308, and 238 loci in the promoter region of the TNFα gene is associated with a shorter onset time of VAP compared with patients carrying only wild-type alleles. It is speculated that the effect of SNPs on the susceptibility of early VAP may be related to the effect of congenital immune response. TNFα SNP alleles tend to produce more proinflammatory cytokines. However, the carrying of allele A was not associated with the severity of VAP (Kotsak et al., 2012). In pulmonary infection, the triggering receptor expressed in myeloid cells (TREM-1) is the key mediator to activate the local inflammatory response. A study reported that SNP in exon 2 (A-T) of TREM-1 gene is associated with an increased risk of VAP in burn patients. Multivariate regression analysis showed that carrying TREM-1T allele was associated with a 3-fold increase in VAP risk. As the number of T alleles increased, the risk of pneumonia increased in a dose-dependent manner. T allele carrying is an independent risk factor for VAP (Rivera-Chávez et al., 2013). Autophagy is a highly conservative mechanism in eukaryotic cells, involving homeostasis and elimination of intracellular pathogens. One of the major proteins involved in autophagy formation and regulation is autophagy-related 16-like 1 (ATG16L1). Non-synonymous SNP of ATG16L1 gene results in autophagy dysfunction. A study has shown that carrying the SNP A allele of ATG16L1 gene can cause autophagy impairment, leading to increased susceptibility to septic shock and organ failure in VAP patients. This is probably due to the fact that autophagy is an important innate mechanism for activating bacterial direct clearance. Carrying ATG16L1 allele A is prone to cause cytokine response deficiencies in circulating monocytes (Savva et al., 2014). The occurrence of VAP may not only be related to the above gene polymorphisms, but also the result of the interaction of multiple genes. It is worth mentioning that early genetic identification of patients can help us to formulate strategies to reduce the risk of VAP in these high-risk groups.

## Other Factors

In addition to the above risk factors which have been extensively studied, Liu et al. (2017) have shown that smoking was the strongest predictor of the development of VAP. They found that the incidence of VAP in smoking patients was 4.37 times that of VAP in non- smoking or smoking cessation patients. The reason may be that long-term smoking leads to impaired pulmonary macrophage function resulting in decreased bacterial clearance, which makes the lungs vulnerable to pathogenic bacteria attack. A single-center prospective study by Papakrivou et al. (2018a, b) identified that intra-abdominal hypertension was an independent risk factor for VAP in ICU patients. Recent studies have shown that intra-abdominal hypertension increases the risk of infection by severely affecting the function of the respiratory system and peripheral organs. Moreover, increased intra-abdominal pressure increases the risk of inhaling gastric contents contaminated by pathogenic microorganisms (Papakrivou et al., 2017, 2018a). A recent single-center retrospective study by Jaffal et al. (2017) found that hyperoxia was an independent risk factor for VAP. This may be due to hyperoxemia leading to denitrification, inhibiting the production of alveolar surfactant, thus promoting atelectasis in mechanical ventilation patients. Moreover, hyperoxemia impairs the migration of alveolar macrophages and the process of phagocytosis, resulting in a weakened bactericidal capacity of alveolar macrophages. However, these findings are not very common in the international world and so they need additional supporting evidence from clinical trials.

## Prevention of VAP

The incidence of VAP in developing countries is much higher than that in developed countries. The reason is that developing countries do not have a good preventive strategy for VAP (Xie et al., 2018). It is very important to identify the risk factors of VAP for guiding the clinical prevention of VAP. At present, VAP preventive measures for the above risk factors have been carried out, such as considering Non Invasive Positive Pressure Ventilation (NIPPV) in some types of respiratory failure, spontaneous breathing trial and daily awakening trial to reduce mechanical ventilation time and hospitalization time; Head of bed elevation to 30–45 degrees, reducing unnecessary sputum suction and reducing the replacement of ventilatory circuit to reduce aspiration; Selective decontamination of the digestive tract and selective oropharyngeal decontamination to reduce the colonization of drug-resistant bacteria (Vazquez Guillamet and Kollef, 2018). For burn patients, fluid infusion should be limited to reduce infection (Luo and Guo, 2015). Although studies have shown that long-term prophylactic use of antibiotics can lead to changes in pathogen resistance, other studies have confirmed that early (<7 days) empirical use of antibiotics in ICU can reduce the incidence of VAP (Dahyot-Fizelier et al., 2018; Evans et al., 2018). A study has shown that β-lactam antibiotics, such as piperacillin-tazobactam, can reduce the incidence of early VAP, but have little effect on the incidence of late VAP (Mirtalaei et al., 2019). Oral care can reduce the reproduction of oral and pharyngeal colonization bacteria. Oral disinfection with 2% chlorhexidine is an effective method to prevent the occurrence of VAP and reduce oral colonization, especially to reduce Gram-positive bacteria (Zand et al., 2017). In addition, the use of probiotics can reduce ICU admission time and hospitalization time of VAP patients, but the use of probiotics has no significant effect on bacteria causing diarrhea, stomach and oropharynx colonization, nor on the incidence of multi-drug resistant pathogens (Mahmoodpoor et al., 2019). In fact, the above preventive measures are often used together clinically, called VAP prevention bundle. VAP prevention bundle requires not only multi-preventive measures to be used together, but also requires medical staff and patients to carry out safety education in order to better improve the operational ability of medical staff and patients’ compliance (Álvarez-Lerma and Sánchez García, 2018). Specific preventive measures for the occurrence of various risk factors of VAP are listed in Table 1.

## Summary

There are various risk factors for VAP occurrence. Each factor is not only an independent risk factor of VAP, but also has an influence on each other. Due to the decline of the physiological and immune functions, elderly patients often have more than one comorbidity, which can lead to an increase in hospital length of stay and mechanical ventilation time, increasing the susceptibility to VAP. The hospital length of stay and the time of mechanical ventilation were significantly prolonged in patients with disorders of consciousness. With the prolongation of hospital stay, invasive procedures in the ICU are also relatively increased, increasing the exposure of patients to the bacterial environment. Therefore, the chance of VAP occurrence has increased greatly. The independence and interaction of these factors have contributed to the occurrence and development of VAP. To identify the risk factors of VAP has a very important guiding role in clinical prevention. In this review, we searched either retrospective or prospective international clinical trials on risk factors for VAP which were published in recent years and performed in a single center or multicenter all over the world. It should be noted that these clinical trials had differences in the sample size of the study, age/sex ratio of enrolled cases, ethnicity and geography, which can lead to inconsistencies in risk factor analysis. In addition, these studies do not have unified diagnostic criteria and treatment measures for VAP, which makes the results lack accuracy. Therefore, further studies with larger sample size and standard definitions for VAP are needed to better understand the global epidemiological characteristics of VAP. Only in this way can we provide more reliable support for better prevention and control of VAP.

## Author Contributions

DW and CW drafted the initial manuscript. All authors developed the concept and design of the study and participated in critical revisions of the manuscript.

## Conflict of Interest Statement

The authors declare that the research was conducted in the absence of any commercial or financial relationships that could be construed as a potential conflict of interest.
